# Pregnant Adults’ Interest in Physical and Mental Health Behavior Change Interventions Using Mobile Technology

**DOI:** 10.1007/s41347-025-00529-8

**Published:** 2025-05-23

**Authors:** Natalie M. Yarish, Cassidy M. Sandoval, Alicia Moulder, Meredith I. Turner, Danielle Symons Downs, Kristin E. Heron

**Affiliations:** 1 School of Exercise Science, College of Health Sciences, Old Dominion University, Norfolk, VA, USA; 2 Department of Psychology, College of Arts and Sciences, Appalachian State University, Boone, NC, USA; 3 Department of Kinesiology, College of Health and Human Development, Pennsylvania State University, University Park, PA, USA; 4 Psychology Department, College of Sciences, Old Dominion University, Norfolk, USA

**Keywords:** Pregnancy, Cardiovascular health, Intervention development, Health behaviors, Mobile health

## Abstract

Improving the health and well-being of pregnant adults is an important public health priority in the USA, and mobile health (mHealth) interventions may provide a scalable strategy to disseminate behavior change programs to pregnant adults. This study assessed pregnant people’s use of mobile technology, interest in making health behavior changes during and after pregnancy, and their willingness to use mHealth interventions. Participants were 133 pregnant adults (*M*_age_ = 27.86; 81% White) living across the USA recruited from social media to complete an online survey. Measures assessed mobile technology use and beliefs about improving mental/physical health in pregnancy and postpartum and using mHealth technology to address such changes. Participants endorsed daily smartphone use, and most participants endorsed wanting to improve their physical and mental health during pregnancy and postpartum. Key areas that emerged from their responses were to improve physical (healthy eating, physical activity, weight control, sleep, hydration) and mental (stress management, reducing depression/anxiety) health. Participants generally thought mobile technology could be a helpful and feasible tool for improving their health. Overall, findings support and extend past research among preconception adults suggesting mobile technology may be a helpful tool for implementing pre- and postnatal health behavior change programs. These findings may also be useful for developing community-informed health behavior interventions for pregnant adults that utilize mHealth approaches. Though these results show promise for the use of mHealth interventions during the perinatal period, feasibility and effectiveness studies are needed to better understand the role of mobile technology in perinatal health promotion.

Improving physical and mental health of pregnant people is an important public health priority in the USA. Cardiovascular disease (CVD) is the leading cause of death in pregnant adults (Benjamin et al., 2017) and accounts for 26.5% of US pregnancy-related deaths (Creanga et al., 2017). Adherence to healthy dietary patterns and increasing physical activity (Parikh et al., 2021) may decrease the risk of CVD among pregnant adults. Less than a quarter of US pregnant adults engage in the recommend amount of exercise during pregnancy and gain the recommended amount of weight during pregnancy (Moore Simas et al., 2013), while 47.2% gained excessive weight and 20.9% inadequate weight gain (Deputy et al., 2015). Additionally, smoking and alcohol use (Biddinger et al., 2022) have been shown to increase the risk of CVD. Approximately 4.6% of pregnant adults reported smoking during pregnancy (Martin et al., 2023) and 14% reported drinking alcohol (CDC, 2022). Poor mental health may also increase the risk of CVD during pregnancy. For example, anxiety and depression have been shown to increase the risk of preeclampsia among pregnant adults (Kurki et al., 2000). In the general population, depression (Bradley & Rumsfeld, 2015; Cohen et al., 2015; Hare et al., 2014), and to a lesser extent, anxiety, and stress (Cohen et al., 2015; Tully et al., 2016) are risk factors for CVD. Thus, community-informed behavior change interventions that focus on improving the broader physical and mental health of pregnant adults are important for reducing the risk of CVD across the life course.

Most existing healthy lifestyle interventions for pregnant adults aim to reduce gestational weight gain (GWG) and gestational diabetes mellitus (GDM), with less focus on CVD. The lifestyle interventions that do exist for GWG and GDM target physical activity and diet behaviors and often do not consider mental health. Systematic reviews and meta-analyses of primarily in-person interventions show reduced GWG (Currie et al., 2013; Gardner et al., 2011; Sui et al., 2012), while other reviews show inconclusive or no effect of improvements in physical activity and diet among pregnant adults (Ronnberg & Nilsson, 2010; Thangaratinam et al., 2012). One systematic review showed that one-third of the multimodal mobile health (mHealth) interventions that combined an app or text messaging, along with social media and email, was correlated with less overall GWG and improved physical activity (Hussain et al., 2020). MHealth interventions have been designed and tested for reducing GWG and GDM and have generally found small, but limited effects on GWG (Downs et al., 2021; Leonard et al., 2021; Sandborg et al., 2021) and GDM (Kennelly et al., 2018) during pregnancy, and conflicting evidence regarding changes in healthy eating behaviors and physical activity. There is far less known regarding mobile lifestyle interventions including mental health for pregnant adults (Zuccolo et al., 2021), highlighting an opportunity for exploring the need for mobile interventions addressing stress, anxiety, and depression during and after pregnancy in addition to physical health behaviors to reduce CVD. However, mHealth interventions have been shown to positively impact cardiovascular health such as enhancing physical and mental health among the general population (Cruz-Cobo et al., 2022), which is promising for the pregnant adults.

Mobile technology offers one option for delivering health behavior interventions, particularly for those of childbearing age 18 to 55 years old who are more likely to be digital natives. There is a growing interest in using mobile technology for behavior change programs before pregnancy (e.g., Materia et al., 2018) and during pregnancy targeting GWG and GDM (e.g., Downs et al., 2021; Kennelly et al., 2018; Leonard et al., 2021; Sandborg et al., 2021). Yet, there has been less emphasis on using mobile technology to target CVD-related factors such as eating behaviors, physical activity levels, substance use, and mental health. Before developing these interventions, it is important to first assess whether pregnant adults are interested in such mobile lifestyle interventions. Further, it is important to determine if pregnant adults are open to making specific health-related behavior changes.

Given the limited research available examining pregnant adult’s willingness to engage in mobile physical and mental health interventions, it is important to research the needs and preferences of pregnant adults in order to develop tailored health behavior interventions. The present study was guided by the Theory of Planned Behavior (Ajzen, 1991) to understand adult’s intentions and beliefs regarding health behavior change, and the Technology Acceptance Model (Davis, 1989; Davis et al., 1989) to understand factors that impact adult’s usage of mobile technology for health behavior change. In brief, the Theory of Planned Behavior posits that people’s attitudes, subjective norms (i.e., perceptions of the behavior), and perceived behavioral control (i.e., perceived ease of engaging in the behavior) influence their intention to engage in a behavior and, in turn, their actual behavior (Ajzen, 1991). The Theory of Planned Behavior informed our development of questions regarding both the target physical and mental health behaviors, as well as behavior of mobile technology use. Previous work among pregnant and postpartum people has concluded that some of the underlying constructs of the Theory of Planned Behavior supported positive behavior change, including behavioral intentions and subjective norm (Downs & Hausenblas, 2007; Symons Downs & Ulbrecht, 2006). In addition, the Technology Acceptance Model supplemented our understanding of technology use. This model posits that perceived usefulness and perceived ease of use influence people’s behavioral intentions to use a technology and in turn their actual use (i.e., behavior; Davis, 1989). These theories were used to assess pregnant adults’ beliefs around making health behavior changes during and after pregnancy, specifically beliefs about utilizing mobile technology to track and improve physical and mental health. Understanding beliefs around such behavior change is useful when designing interventions. These beliefs can inform decisions around the behaviors adults see as most important to change (e.g., what they are most motivated to change), the optimal type(s) of technology to use (e.g., smartphones, tablets, traditional phones), and format of intervention delivery (e.g., app, text message, websites).

The goal of this study was to assess pregnant adult’s beliefs about making health behavior changes during and after pregnancy and their beliefs and willingness to use mobile technology interventions to make such changes. Smartphones are widely used for a variety of purposes (ConsumerAffairs*^®^*, 2024), and health behavior change apps (McKay et al., 2019) and pregnancy apps (Hughson et al., 2018; Lupton & Pedersen, 2016; Wang et al., 2019) are broadly available. Thus, we predicted that adults would generally be open to the use of mobile technology for physical and mental health behavior change interventions. Consistent with past research that similarly examined mobile technology preferences among pre- and inter-conception adults (Downs et al., 2019), we did not make specific predictions about the types of behaviors pregnant adults would want to change or specific beliefs about mobile technology use for behavior change.

## Method

### Participants

Participants were 133 pregnant adults (*M*_*age*_ = 27.86, *SD*_*age*_ = 4.84) recruited via social media for participation in an online study about health behaviors and interest in mHealth interventions for pregnancy. Participants were eligible to participate if they endorsed being at least 18 years or older, being assigned female at birth, as well as being currently being pregnant. Initially, 189 responses were collected, and from this, 6 were removed for duplicate responding (based on IP address), 28 were removed for random responding or suspected spam/bot responding (e.g., implausible due dates, weeks of pregnancy, weight, etc., nearly duplicate responses in very short timeframe, etc.), and 22 were removed for failing to respond to a majority of the survey items. Of the final sample of 133 participants, the majority were White (*n* = 108, 81.2%) and non-Hispanic (*n* = 117, 88.0%). Approximately half the sample were women who have never given birth to a live child (*n* = 69, 51.9%). A vast majority of participants (*n* = 128, 96.2%) reported their current pregnancy was a single pregnancy. See Table 1 for additional demographic information.

### Measures

#### Demographics and Pregnancy Health Background

A total of 17 questions created by the research team were used to gather demographic and pregnancy-specific information about participants including age, race, ethnicity, sexual orientation, self-reported gestational weeks, and a history of previous pregnancies. Self-reported pre-pregnancy and current height and weight were used to calculate preconception and current body mass indices.

#### Desire to Improve Physical and Mental Health

Participants were asked to indicate the degree to which they desired to improve their physical and mental health (e.g., “I would like to improve my physical health”) using a 7-point Likert scale (1 = *Strongly Disagree* and 7 = *Strongly Agree*). Participants were also asked to endorse specific domains of physical and mental health they wished to improve either during or after pregnancy. Options included increase physical activity, eat healthier, manage weight, improve sleep, drink more water, reduce stress, reduce anxiety or depression, reduce alcohol consumption, and reduce or quit smoking. These items were developed by the researchers for the purpose of this study.

#### Mobile Technology Use

Eight items assessed typical smartphone usage habits, including the type of smartphone used (e.g., Apple or Android), services available on current service plan (e.g., text messaging, data), and frequency with which smartphones were used for various purposes (e.g., send/receive texts, browse the internet, use game or social media apps), including previous usage of health or lifestyle apps to track or change health habits. Items assessing mobile technology use were adapted from Heron, Romano, and Braitman (2019).

#### Technology and Health

We created questions based on items used in previous studies (Cramer et al., 2022; Romano et al., 2022) and informed by the Theory of Planned Behavior and Technology Acceptance Model to assess participants’ perceptions of mHealth using a 7-point Likert scale. Eight of these items assessed interest in, willingness to, and beliefs about utilizing mobile technology to track and improve physical and mental health (1 = *Strongly Disagree* and 7 = *Strongly Agree*). Five of these items assessed the extent to which participants believed that mobile technologies targeting health changes would be useful, enjoyable, interesting, important, and efficient to use with a 7-point scale (1 = *Not at All* and 7 = *Very Much*). Participants also indicated whether they would be interested in participating in future programs targeting physical or mental health if it were at no cost to them or their insurance (yes/no).

### Procedures

Participants were invited to participate via social media postings. Interested individuals clicked the survey link and answered brief study eligibility questions to determine they met inclusion criteria for the study. The survey was programmed to automatically determine eligibility, and eligible participants were automatically forwarded to the study survey and instructed to review an online consent form. Informed consent was obtained from all individual participants. They then completed an online survey. Compensation for participation was provided in the form of a $10 electronic gift card. The Old Dominion University Institutional Review Board approved all study procedures, which were in accordance with the ethical standards of the 1964 Helsinki Declaration and its later amendments. Prior to data analyses, all responses were examined for completeness and authenticity; responses that were incomplete or appeared automated/spam were removed. All data management and analyses were conducted in SPSS 27 (SPSS, 2020).

## Results

### Mobile Technology Use

Nearly all participants (*n* = 127, 96.9%) reported using a smartphone on a daily basis. Results regarding the various purposes and frequencies of smartphone use were reported by participants and are presented in Table 2. Mobile technology appeared to be accessible to the study sample, as most participants endorsed having a service plan that allowed unlimited phone calls (68.4%), text messaging (57.9%), and data (92.5%). Additionally, participants generally reported mobile technology to be easy to use (*M* = 5.85, *SD* = 1.30) and affordable (*M* = 5.73, *SD* = 1.21).

### Desired Physical and Mental Health Changes

**Fig. 1:**
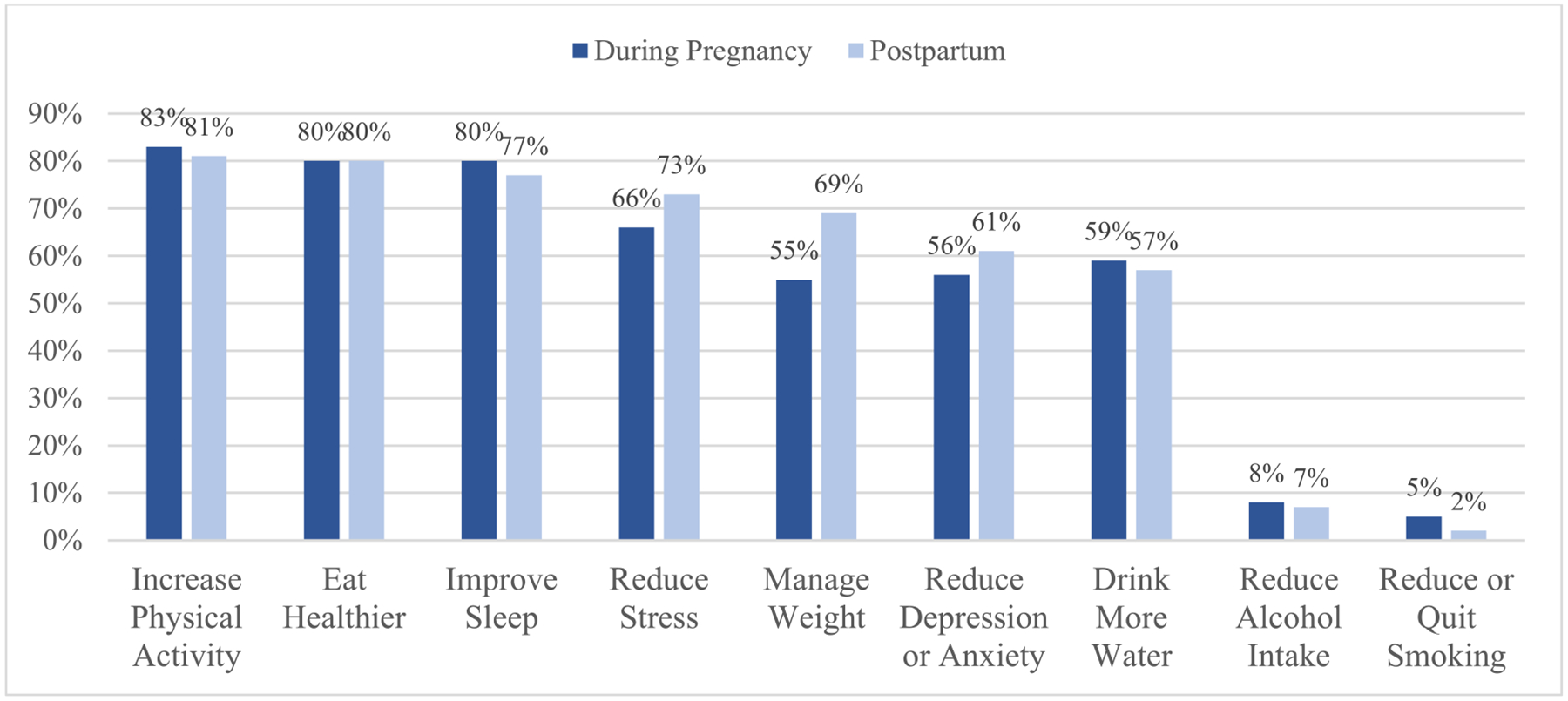
Percentage of women interested in addressing various aspects of health (*n* = 127)

Participants showed interest in improving both mental (*M* = 5.85, *SD* = 1.26) and physical (*M* = 5.98, *SD* = 1.43) health. Percentages of the sample that endorsed various domains of physical and mental health they wished to improve during pregnancy and postpartum are presented in Fig. 1. The majority of participants were interested in increasing physical activity, eating healthier, and improving sleep during pregnancy and postpartum, while few participants reported interest/desire in reducing alcohol (7.5%) or tobacco (4.5%) use. More participants were interested in reducing stress, anxiety, and depression and managing weight postpartum, rather than during pregnancy (see Fig. 1).

### Interest in Using Mobile Technology

Pregnant adults generally reported being willing to use mobile technology to track (*M* = 5.81, *SD* = 1.44) and change (*M* = 5.44, *SD* = 1.39) health behaviors. There was a general belief that use of mobile technology could improve emotional (*M* = 5.17, *SD* = 1.34) and physical (*M* = 5.52, *SD* = 1.26) well-being, and many pregnant adults reported thinking they would be successful in using mobile technology to improve their health (*M* = 5.26, *SD* = 1.38). Participants reported that having a pregnancy-related health behavior change program available on a smartphone would be useful (*M* = 5.83, *SD* = 1.42), enjoyable (*M* = 5.29, *SD* = 1.30), interesting (*M* = 5.50, *SD* = 1.45), important (*M* = 5.36, *SD* = 1.33), and efficient (*M* = 5.57, *SD* = 1.28). The vast majority of participants (*n* = 122, 91.7%) endorsed interest in participating in future programs focused on improving physical or mental health surrounding pregnancy.

## Discussion

Community-informed lifestyle intervention development can lead to increasing deeper and more nuanced understandings of desired health behaviors (Irby et al., 2021) that are more likely to be effective and sustained, and ultimately improving the cardiovascular health of pregnant adults. Findings from our study showed that during pregnancy, adults are interested in interventions that focus on increasing physical activity, eating healthier, and improving sleep during their pregnancy and postpartum. Thus, the perinatal period may be a critical time to implement behavior change interventions to reduce the risk of CVD. Few pregnant adults reported needing interventions to reduce alcohol or tobacco during pregnancy and postpartum. During postpartum specifically, adults report needing interventions that focus on reducing stress, anxiety, and depression and managing weight.

The findings from the current study extend existing research on developing behavior change interventions during the preconception period and more generally during reproductive years. For example, previous research identified key factors (i.e., nutrition, physical activity, stress, substance use, smoking in the home, and infection) in the first phase of an in-person behavior change intervention designed to promote adult’s pre-conceptional health in rural, low-income communities (Downs et al., 2009; Hillemeier et al., 2008; Weisman et al., 2006). Additionally, research has found that intervention components should be targeted based on reproductive stage (i.e., preconseptional, interconceptional, and postconceptional; Weisman et al., 2006). Participants in our current study extended these findings by identifying key heart healthy behavior change areas (i.e., healthy eating, physical activity, weight control, sleep, hydration, stress management, reducing depression/anxiety) that would be beneficial for them during and after pregnancy that could be implemented via mobile intervention.

Pregnant adults identified that using technology in digital behavior change interventions could improve their emotional, physical, and mental health during pregnancy and postpartum. The majority of pregnant adults reported behavior change interventions delivered through mobile technology would improve their cardiovascular health. This is consistent with a recent systematic review and meta-analysis, which recommended that traditional lifestyle interventions should be replaced by creative concepts designed for specific needs of pregnant adults (Behnam et al., 2022), citing that smartphone applications can support behavior change interventions (van Dijk et al., 2020). Additionally, interventions focused on lowering prenatal GWG that incorporated technology and face-to-face sessions, tracking tools, and wearable devices daily were associated with a higher magnitude of effect on improving physical activity and healthy eating behaviors (Leonard et al., 2021). Most pregnant adults in our study reported using mobile technology more than once a day to receive text messages, emails, internet use, general apps, and lifestyle-related/health specific apps. The findings from our study coupled with the findings form Leonard and colleagues (2021) support the value of implementing technology-supported interventions daily. However, future implementation work should examine whether pregnant adults become habituated to the daily technology-supported interventions, which may weaken the effect of the intervention.

Findings support and extend past research among preconception adults suggesting mobile technology may be a helpful tool for implementing health behavior change programs around pregnancy (e.g., Downs et al., 2019), as this sample of pregnant adults demonstrated interest and willingness to use mobile technology to track and change health behaviors during and after their pregnancy. Previous qualitative research shows that pregnant adults would like to incorporate health applications within their daily routine (Goetz et al., 2017). Our findings extend this research by identifying key areas where pregnant adults are interested in using mobile technology to improve their physical (healthy eating, physical activity, weight control, sleep, hydration) and mental (stress management, reducing depression/anxiety) health. We also learned about areas of least interest. Only a few pregnant adults were interested in smoking cessation and alcohol use reduction as part of a lifestyle intervention. However, this could be a product of fewer pregnant adults smoking or using alcohol during pregnancy. It may still be important to target these health behaviors among adults who are smoking or drinking alcohol during pregnancy. These findings also highlight specific time periods within the perinatal period pregnant adults may want to focus on cardiovascular health improvements. For example, in our sample, more pregnant adults endorsed interest in reducing stress, managing weight, and reducing depression or anxiety during the postpartum period than during pregnancy.

We acknowledge the challenges that accompany designing and implementing interventions during the perinatal period which can involve pregnancy-related side effects and physical limitations. However, our study and previous research show that pregnant adults want mHealth lifestyle interventions to improve physical and mental health. Given the transitional nature of the pregnancy and postpartum periods, more research is needed to understand how to effectively target pregnant adult’s health behaviors of interest (i.e., healthy eating, physical activity, weight control, sleep, hydration, stress management, and reducing depression/anxiety).

## Limitations

This study is not without limitations. Our sample was 81% White, which limits the generalizability of the findings to adults of other ethnic/racial groups. This is similar to other studies examining health outcomes during the perinatal period (Goyal et al., 2023). A lack of racial representation in perinatal research contributes to the perpetuation of racial health disparities, and it is important for future studies to do more targeted recruitment of racial minority individuals. Future research should engage in targeted recruitment of racial minority pregnant adults via social media or online recruitment firm panels (either through paid advertisements or by targeting groups that have greater racial diversity), or through targeted in-person recruitment (e.g., via medical offices) to increase representation of non-White pregnant adults. We did not examine socioeconomic status or education level among the sample. Although we assessed interest/desire in reducing alcohol or tobacco use in a behavior change intervention, we did not assess substance use among our sample. Thus, for both socioeconomic and substance use, we are unable to describe these characteristics and health behaviors among our sample. We also acknowledge that this was a cross-sectional, self-report survey and that willingness to use technology or ask for specific interventions, although an important part of the behavior change process (Ajzen, 1991; Davis, 1989), may not necessarily translate into actual uptake of these interventions. Although 97% of US adults have a smartphone (Pew Research Center, 2023), not all have ongoing, sustained access, which may impact attrition rates and success of a potential behavior change intervention delivered through mobile technology. We recruited our convenience sample through social media, which suggests this group of pregnant adults could be more likely to have and use mobile technology. Furthermore, for some of our specific research inquiries, we were not able to use validated measures as they currently do not exist.

## Conclusion

The current study can inform future development of mHealth prenatal and postpartum lifestyle interventions to promote cardiovascular health. Pregnant adults are interested in mHealth interventions targeting physical activity, diet, sleep, and mental health during pregnancy and postpartum. Additionally, implementing mHealth lifestyle interventions via smartphones is a promising approach to changing health behaviors. Additional research on feasibility and effectiveness is needed to inform mHealth health behavior intervention programs for pregnant adults.

## Figures and Tables

**Table 1:** Demographic characteristics of participants (*n* = 127)

	*n*	%
Trimester		
First	31	23.3
Second	45	33.8
Third	57	42.9
Parity		
Nulliparous	69	51.9
Primiparous	38	28.6
Multiparous	26	19.5
Pregnancy type		
Single	128	96.2
Multiple	1	0.8
Not sure	4	3.0
Race		
White	108	81.2
Black	7	5.4
Asian or Asian-American	4	3.0
Middle Eastern or Northern African	2	1.5
Native Hawaiian or Other Pacific Islander	1	0.8
Other	5	3.8
Multiracial	6	4.5
Ethnicity		
Hispanic/Latinx	16	12.0
Non-Hispanic/Latinx	117	88.0
Sexual orientation and identity		
Heterosexual	124	93.2
Bisexual	5	3.8
Queer or gender nonconforming	1	0.8
BMI (*M, SD*)	26.80 (5.81)	
Preconception BMI (*M*, *SD*)	24.41 (6.28)	

**Table 2:** Daily usage of mobile devices (*n* = 127)

	*n*	%
**Send or receive text messages**		
More than once a day	88	66.2
About once a day	13	9.8
A few times a week	24	18.0
A few times a month	5	3.8
A few times a year	0	0
Never or rarely	2	.8
**Access email**		
More than once a day	79	59.4
About once a day	22	16.5
A few times a week	28	21.5
A few times a month	2	1.5
A few times a year	0	0
Never or rarely	0	0
**Access internet**		
More than once a day	100	75.2
About once a day	14	10.5
A few times a week	15	11.3
A few times a month	2	1.5
A few times a year	0	0
Never or rarely	0	0
**Use general apps**		
More than once a day	98	73.7
About once a day	11	8.3
A few times a week	18	13.5
A few times a month	4	3.0
A few times a year	0	0
Never or rarely	0	0
**Use health/lifestyle-related apps**		
More than once a day	47	35.3
About once a day	30	22.6
A few times a week	39	29.3
A few times a month	9	6.8
A few times a year	1	.8
Never or rarely	5	3.8

## Data Availability

The data that support the findings of this study are available on request from the corresponding author, Natalie M. Yarish. The data are not publicly available due to restrictions containing information that could compromise the privacy of research participants.
